# Impact of p16 Status and Anatomical Site in Anti-PD-1 Immunotherapy-Treated Recurrent/Metastatic Head and Neck Squamous Cell Carcinoma Patients

**DOI:** 10.3390/cancers13194861

**Published:** 2021-09-28

**Authors:** Kate Clancy, Chelsea S. Hamill, W. Quinn O’Neill, Brandon Vu, Jason Thuener, Shanying Gui, Shawn Li, Nicole Fowler, Rod Rezaee, Pierre Lavertu, Jay Wasman, Monaliben Patel, Hira Shaikh, Eric Vick, Anant Madabhushi, Trisha M. Wise-Draper, Kyunghee Burkitt, Theodoros N. Teknos, Quintin Pan

**Affiliations:** 1 Department of Otolaryngology-Head and Neck Surgery, University Hospitals Cleveland Medical Center, Case Western Reserve University School of Medicine, Cleveland, OH 44106, USA; kate.clancy@uhhospitals.org (K.C.); chelsea.hamill@uhhospitals.org (C.S.H.); wqo@case.edu (W.Q.O.); jason.thuener@uhhospitals.org (J.T.); shawn.li@uhhospitals.org (S.L.); nicole.fowler@uhhospitals.org (N.F.); rod.rezaee@uhhospitals.org (R.R.); pierre.lavertu@uhhospitals.org (P.L.); theodoros.teknos@uhhospitals.org (T.N.T.); 2 University Hospitals Seidman Cancer Center, Cleveland, OH 44106, USA; brandon.vu@uhhospitals.org (B.V.); Shanying.gui@uhhospitals.org (S.G.); jay.wasman@uhhospitals.org (J.W.); Monaliben.patel@uhhospitals.org (M.P.); kyunghee.burkitt@uhhospitals.org (K.B.); 3 Department of Pathology, University Hospitals Cleveland Medical Center, Case Western Reserve University School of Medicine, Cleveland, OH 44106, USA; 4 Department of Medicine, University Hospitals Cleveland Medical Center, Case Western Reserve University School of Medicine, Cleveland, OH 44106, USA; 5 Department of Internal Medicine, Division of Hematology/Oncology, University of Cincinnati, Cincinnati, OH 45267, USA; shaikhhl@ucmail.uc.edu (H.S.); vickec@ucmail.uc.edu (E.V.); wiseth@ucmail.uc.edu (T.M.W.-D.); 6 Department of Biomedical Engineering, Case Western University School of Engineering, Cleveland, OH 44106, USA; anant.madabhushi@case.edu; 7 Louis Stokes Cleveland Veterans Administration Medical Center, Cleveland, OH 44106, USA; 8 Case Comprehensive Cancer Center, Case Western Reserve University School of Medicine, Cleveland, OH 44106, USA

**Keywords:** anti-PD-1 therapy, checkpoint inhibition, head and neck squamous cell carcinoma, immunotherapy, and p16

## Abstract

**Simple Summary:**

Anti-PD-1 immunotherapies are approved for head and neck squamous cell carcinoma in the recurrent/metastatic setting, and utilization of these high-cost biologics is expected to increase as other indications are approved. Due to the high cost and selective response rate of these immunotherapy biologics in HNSCC, it is imperative to better define which patient subsets will realize a clinically meaningful benefit with anti-PD-1 treatment. The impact of anatomical site and p16 status on the efficacy of anti-PD-1 inhibitors remains unresolved. We showed that anatomical site and p16 status are associated with overall survival in anti-PD-1-treated HNSCC patients from a single-institution, real-world cohort.

**Abstract:**

In head and neck squamous cell carcinoma (HNSCC), anti-PD-1 inhibitors are approved for recurrent/metastatic (R/M) disease and anticipated to expand to other indications. The impact of p16 status and anatomical site on overall survival (OS) in immunotherapy-treated HNSCC patients remains unresolved. We performed a retrospective analysis of R/M HNSCC patients receiving anti-PD-1 immunotherapy at our academic medical center with an extensive community satellite network. Fifty-three R/M HNSCC patients were treated with anti-PD-1 immunotherapy and had a median OS of 6 months. Anatomical site was associated with distinct OS; oropharynx and larynx patients have superior OS compared to oral cavity patients. Analysis of the OPSCC subset showed p16+ status as a favorable, independent prognostic biomarker (HR 7.67 (1.23–47.8); *p* = 0.029). Further studies to assess the link between anatomical site, p16 status, and anti-PD-1 treatment outcomes in large cohorts of R/M HNSCC patients managed in real-world clinical practices and clinical trials should be prioritized.

## 1. Introduction

About 850,000 new head and neck squamous cell carcinoma (HNSCC) cases are diagnosed each year worldwide [1]. HNSCC patients are primarily managed with definitive chemoradiation or surgical resection of the primary tumor and lymph nodes, followed by adjuvant radiation, with or without platinum-based chemotherapy. Despite the utilization of these multi-disciplinary treatment modalities, ~50% of these patients develop recurrent/metastatic (R/M) disease within 3 years of initial diagnosis [2]. In R/M HNSCC patients without surgical or re-irradiation options, immunotherapy, as a single agent or in combination with platinum-based chemotherapy, is the preferred alternative.

Immunotherapies, particulary checkpoint inhibitors, have shown dramatic clinical responses in numerous solid malignancies including HNSCC [3,4,5,6,7,8]. In 2016, anti-PD-1 immunotherapies, nivolumab and pembrolizumab, were approved in the second-line setting for platinum-refractory, R/M HNSCC patients. More recently, in 2019, pembrolizumab was approved as a first-line treatment option in unresectable, R/M HNSCC patients. PD-L1 immunohistochemistry has demonstrated some value in identifying responders to anti-PD-1 treatment; in fact, single-agent pembrolizumab is indicated as a first-line, single-agent therapy for R/M HNSCC patients whose tumors express PD-L1 with a combined positive score (CPS) ≥ 1 [7,8].

Incidence of human papillomavirus-associated (HPV+) HNSCC, which predominantly originates in the oropharynx, has increased dramatically in recent decades and this trend is expected to continue for the foreseeable future [9]. HPV+ oropharynx squamous cell carcinoma (OPSCC) is diagnosed using p16 immunohistochemistry, and are biologically and clinically distinct from HPV- OPSCC [10]. Patients with p16+ OPSCC generally have superior treatment responses and outcomes to p16- OPSCC patients [11,12,13,14]. The landmark phase III trial, Checkmate 141, suggests that nivolumab may be more active in the p16+ than in the p16- R/M setting; median OS was 9.1 months for p16+ OPSCC patients and 7.5 months for p16- OPSCC patients [7]. The final analysis of another randomized phase III study, KEYNOTE-048, showed that single-agent pembrolizumab is equally superior, with an identical hazard ratio of 0.81, to cetuximab + platinum-based chemotherapy in p16+ and p16- OPSCC [8]. However, neither of these studies were designedor powered to investigate the effects of p16 status on treatment response with these PD-1 inhibitors. Therefore, the impact of p16 status on anti-PD-1 immunotherapies in the OPSCC population remains unresolved.

## 2. Materials and Methods

Our study (IRB# 20191051) was approved by our Institutional Review Board at University Hospitals Cleveland Medical Center. We queried the University Hospitals Seidman Cancer Center composite clinical database and identified R/M HNSCC patients treated with anti-PD-1 immunotherapies, nivolumab and pembrolizumab, from December 2015 to October 2019. Patient chart review was performed as a quality control step to ensure patients’ clinical and treatment histories. Patient age in this study was defined as the age at initiation of anti-PD-1 immunotherapy. Tobacco smoking status was defined as yes if a patient self-identified as a current/former smoker at initial diagnosis or no if a patient had no smoking history. p16 immunohistochemistry (IHC) is a standard-of-care assay for OPSCC primaries at our academic medical center and defined as p16+ if there was strong and diffuse nuclear and cytoplasmic staining in ≥70% of tumor cells. Patients with non-OPSCC primaries are not routinely assessed by p16 IHC due to the low prevalence of HPV in these anatomical sites and were defined as p16- in this study.

All statistical analyses were performed using R (version 4.0.2). Log-rank testing was performed for each group-wise comparison. Continuous parameters are summarized using descriptive statistics, which includes means and standard deviations. All continuous data were analyzed using two-tailed *t*-tests. Categorical parameters are summarized using frequencies and percentages. Categorical variables were compared using Fisher’s exact test for 2 × 2 contingency table and chi-squared test for contingency tables whose dimensionality exceeds 2 × 2. Survival analysis was performed with Kaplan–Meier curves and log-rank tests using the R package survival and plotted by ggplot2. Multivariate logistic regression analysis wfas utilized to create a forest plot. A *p*-value of <0.05 was considered statistically significant.

## 3. Results

### 3.1. Anatomical Site Is Associated with OS in Immunotherapy-Treated R/M HNSCC Patients

We queried our annotated HNSCC clinical database and identified 53 R/M patients treated with single-agent, anti-PD-1 therapy, nivolumab and/or pembrolizumab (Table 1). Twenty patients were treated in the single-line and 33 patients were treated in the second/third lines following some combination of surgical salvage, radiation, and/or chemotherapy. This cohort was primarily managed with nivolumab (71.2%; 37/52). One patient was treated with pembrolizumab, progressed, and then switched to nivolumab; this case was excluded from the nivolumab vs. pembrolizumab analyses. A majority of our R/M cohort presented with distant metastasis (79.2%; 42/53) and self-identified as current/former tobacco users (83%; 44/53). Our study population skewed toward oropharynx (39.6%; 21/53), followed by oral cavity (28.3%; 15/53), larynx (18.9%; 10/53), and other anatomical sites (unknown primary and hypopharynx; 13.2%; 7/53).

**Figure 1:**
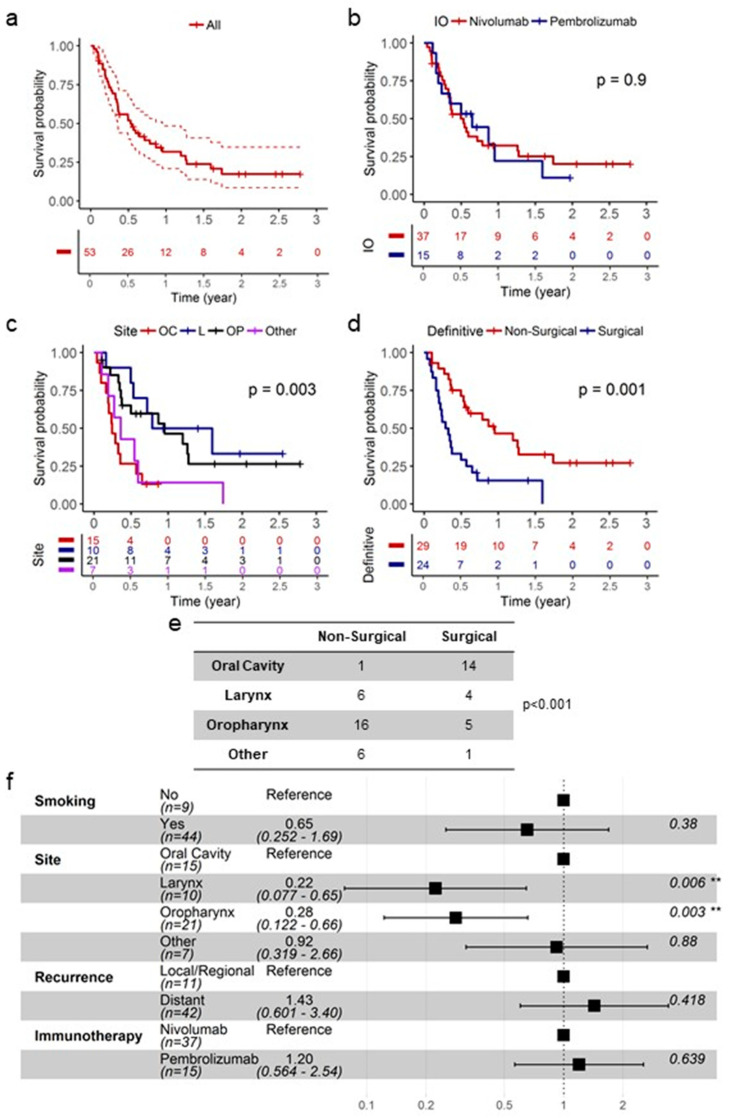
Anatomical site is associated with OS in immunotherapy-treated R/M HNSCC patients. (**a**) KM plot for the entire cohort. (**b**) KM plot based on anti-PD-1 immunotherapy choice. (**c**) KM plot based on anatomical site. (**d**) KM plot based on initial treatment approach. (**e**) Initial treatment approach based on anatomical site. (**f**) Multivariate model for the entire cohort presented as a forest plot. ** *p* < 0.01.

In our R/M HNSCC cohort, median overall survival (OS) after initiating immunotherapy was 6.3 months (Figure 1a). Nivolumab and pembrolizumb showed equivalent efficacy (log-rank, *p* = 0.9) and this observation was maintained even after adjusting for co-variates in a Cox regression model (Figure 1b). Anti-PD-1 immunotherapies were less active in R/M oral cavity SCC patients than in R/M oropharyngeal and laryngeal SCC patients; median OS was 2.9, 11.4, and 14.2 months post-immunotherapy treatment in oral cavity, oropharynx, and larynx SCC, respectively (Figure 1c). As shown in Figure 1d, R/M patients who were surgically managed in the initial treatment setting had inferior prognosis compared to non-surgically managed patients (log-rank, *p* = 0.001), which is likely due to the fact that a majority of the non-surgically managed patients had oropharynx SCC (Figure 1e; 55.2%; 16/29; χ2, *p* < 0.001). Multivariate model, adjusting for smoking, anatomical site, recurrence pattern at presentation (locoregional vs. distant), and immunotherapy choice, revealed that anatomical site is an independent prognostic biomarker in this cohort; oropharyngeal (HR 0.28, 95% CI: 0.122 to 0.66; *p* = 0.003) and laryngeal (HR 0.22, 95% CI: 0.077 to 0.65; *p* = 0.006) SCC patients had superior prognosis compared to oral cavity SCC patients (Figure 1f).

### 3.2. p16+ Is an Independent Prognostic Biomarker in Immunotherapy-Treated R/M OPSCC Patients

**Figure 2:**
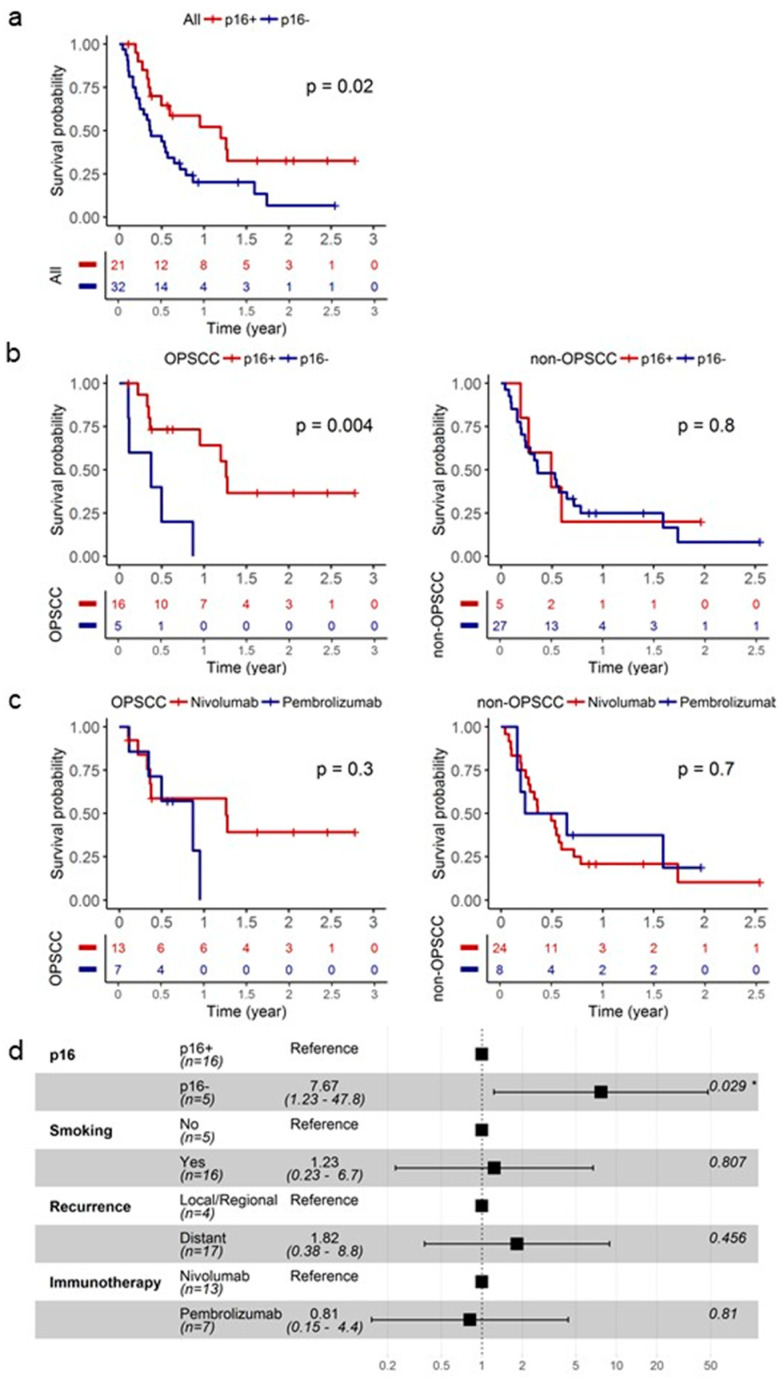
p16+ is an independent prognostic biomarker in immunotherapy-treated R/M OPSCC patients. (**a**) KM plot stratified based on p16 status for the entire cohort. (**b**) KM plot stratified based on p16 status for the oropharynx and non-oropharynx SCC cohorts. (**c**) KM plot stratified based on anti-PD-1 immunotherapy choice for the oropharynx and non-oropharynx SCC cohorts. (**d**) Multivariate model for the oropharynx SCC cohort presented as a forest plot. * *p* < 0.05.

In Figure 2a, analysis of our R/M HNSCC study cohort based on p16 status revealed that p16+ patients had superior OS compared to p16- patients (log-rank, *p* = 0.02). Subgroup analyses showed that the survival advantage conferred to p16+ status was limited to the oropharyngeal SCC (log-rank, *p* = 0.004) patients and was not observed in the non-oropharyngeal SCC (log-rank, *p* = 0.8) patients (Figure 2b). Median OS was 15.1 months and 4.5 months for p16+ and p16- oropharyngeal SCC patients, respectively. Patient characteristics were balanced between the p16+ and p16- R/M oropharyngeal cohorts with the exception of gender; p16+ patients tend to be males (Table 2). Anti-PD-1 immunotherapy choice showed no impact on OS when our R/M cohort was analyzed based on anatomical site (Figure 2c). Cox regression, adjusting for co-variates, revealed p16 status as an independent prognostic biomarker in the immunotherapy-treated R/M oropharyngeal SCC patients; p16- patients had a 7.67-fold (95% CI: 1.23 to 47.8; *p* = 0.029) increase in risk of death compared to p16+ patients (Figure 2d).

## 4. Discussion

Nivolumab and pembrolizumab are two anti-PD-1 antibodies that are indicated for R/M HNSCC. FDA approved these two immunotherapeutic agents in 2016 for R/M HNSCC patients in the second-line setting following progression from platinum-containing regimens. More recently, pembrolizumab, based on phase III clinical trial evidence, was approved as first-line therapy R/M HNSCC in combination with platinum-based chemotherapy or as a single agent in patients with PD-L1-positive (CPS ≥ 1) tumor expression [8]. To the best of our knowledge, pivotal clinical trials with these two immunotherapies did not ascertain if specific head and neck anatomical sites are associated with distinct clinical responses and outcomes. Our results provide an initial signal that anatomical site may matter in immunotherapy response; oral cavity SCC patients are less likely to have durable responses to anti-PD-1 therapies compared to oropharyngeal and laryngeal SCC patients.

HPV+, based on HPV in situ hybridization or p16 IHC, is a favorable prognostic biomarker in OPSCC in response to various treatment approaches, surgical and chemotherapeutics, in the locally advanced and R/M settings [11,12,13,14]. In light of these clinical studies and the notion that HPV+ tumors have an intrinsic and persistent source of viral antigens to illicit an anti-tumor immunity response, it was anticipated that anti-PD-1 immunotherapies may be more active in p16+ than in p16- R/M OPSCC patients. The results from phase I and II trials with pembrolizumb were mixed in R/M OPSCC patients; some studies showed that p16+ status was associated with an improved response rate, while another study reported similar clinical benefit between p16+ and p16- patients [5,15,16]. Unfortunately, results from the two multi-institutional phase III trials with pembrolizumab (KEYNOTE-048) or nivolumab (CheckMate 141) did not provide sufficient clarity to ascertain the impact of p16 status on the OS of R/M OPSCC patients treated with these PD-1 blocking agents [7,8]. Our single institutional study, with a heterogeneous patient population, showed that p16+ status is an independent prognostic biomarker in immunotherapy-treated R/M OPSCC patients; p16- patients had a 7.67-fold increase in risk of death compared to p16+ patients. In light of our data, post-hoc analyses of KEYNOTE-048 and CheckMate 141 to compare OS of p16+ and p16- OPSCC patients treated with anti-PD-1 antibodies should be performed to determine if our real-world cohort finding will be validated in a well-defined, homogenous clinical trial population.

HPV/p16 status, tobacco smoking, N-stage, and T-stage are the major determinants of OS in OPSCC and these four variables can be used as risk classifiers in this population [11]. HPV+ OPSCC patients are classified as low-risk and intermediate-risk categories in the definitive setting based on tobacco smoking history and N-stage; low-risk patients are non-smokers (≤10 pack-years) or smokers (>10 pack-years) with N0-N2a, whereas intermediate-risk patients are smokers with N2b-N3 [11]. HPV- OPSCC patients are at high risk, with the exception of a small subset of non-smoker individuals with T2-3 tumors, which are considered intermediate risk [11]. In our OPSCC study cohort, 76% (16/21) were self-reported smokers (current or former) and this proportion was similar regardless of p16 status, which was 75% (12/16) and 80% (4/5) for p16+ and p16- patients, respectively. The predominance of smokers in our OPSCC subset was anticipated, since HPV+ smokers are at intermediate-risk, and thus have a higher probability of progressing with R/M disease. Tobacco smoking history was not an independent prognostic biomarker in this OPSCC subset, suggesting that tumor mutational load, recognized to be higher in smokers than non-smokers, may not be a robust biomarker of immunotherapy response in the R/M OPSCC setting.

Pembrolizumab and nivolumab are designed to work against the same target, PD-1, suggesting that these therapeutics may have equivalent efficacies. This assumption appears to hold true in melanoma, as OS was not statistically different between patients treated with pembrolizumab and nivolumab [17]. In contrast, data in non-small cell lung carcinoma provide some evidence that these two immunotherapies may not be equivalent in efficacy [18,19]. In our real-world cohort, the multivariable regression model showed that pembrolizumab and nivolumab have comparable activities in the entire HNSCC cohort as well as the OPSCC subset. To our knowledge, this is the first study to compare the efficacies of these two anti-PD-1 immunotherapies in R/M HNSCC.

We acknowledge several limitations of our work including retrospective design, single institutional experience, and small sample size, which precludes exploration of patient selection biases, such as definitive and salvage treatment histories and performance status.

## 5. Conclusions

Anti-PD-1 therapy utilization is expected to increase in HNSCC as indication expands beyond the R/M setting. Due to the high cost and selective response rate of these immunotherapy biologics, it is imperative to better define which HNSCC populations, whether based on anatomical site and/or molecular markers, will realize clinically meaningful benefit with anti-PD-1 treatment compared to other, more cost-effective treatment regimens in order to limit the financial toxicity to our health system. A concerted effort to further explore the interactions between p16 status and anatomical site, and treatment outcomes in large multi-institutional cohorts of real-world and clinical trial patients managed with anti-PD-1 biologics should be prioritized and may reveal patient subsets that are exceptional and poor responders.

## Figures and Tables

**Table 1:** Clinicopathologic characteristics of R/M HNSCC patients treated with anti-PD-1 immunotherapy.

**Age, years (range)**	63.4 (23–86)
**Gender**	
Male	75% (*n* = 40)
Female	25% (*n* = 13)
**Smoking history**	
Yes	83% (*n* = 44)
No	17% (*n* = 9)
**Anatomical Site**	
Oropharynx	40% (*n* = 21)
Oral Cavity	28% (*n* = 15)
Larynx	19% (*n* = 10)
Other	13% (*n* = 7)
**Definitive treatment**	
Non-Surgical	55% (*n* = 29)
Surgical	45% (*n* = 24)
**Time to recurrence** (months after definitive treatment)	7 (1–57)
**Recurrence**	
Local/Regional	21% (*n* = 11)
Distant	79% (*n* = 42)
**Immunotherapy**	
Pembrolizumab	28% (*n* = 15)
Nivolumab	70% (*n* = 37)
Both	2% (*n* = 1)

**Table 2:** Clinicopathologic characteristics of R/M OPSCC patients treated with anti-PD-1 immunotherapy.

Clinicopathologic Characteristics	p16+ (*n* = 16)	p16- (*n* = 5)	*p* Value
Age	65	58	0.23
Gender Male Female			
	94% (*n* = 15) 6% (*n* = 1)	40% (*n* = 2) 60% (*n* = 3)	0.03
Smoking History Yes No	75% (*n* = 12) 25% (*n* = 4)	80% (*n* = 4) 20% (*n* = 1)	1
Anatomical Site Tonsil/Oropharynx Base of Tongue	69% (*n* = 11)	60% (*n* = 3)	1
	31% (*n* = 5)	40% (*n* = 2)	
Definitive treatment Non-surgical Surgical	88% (*n* = 14)	40% (*n* = 2)	0.06
	12% (*n* = 2)	60% (*n* = 3)	
Time to recurrence (months after definitive treatment)	17 (1–57)	7 (1–26)	0.21
Recurrence Local/Regional Distant	12% (*n* = 2) 88% (*n* = 14)	60% (*n* = 3) 40% (*n* = 2)	0.23
Immunotherapy Pembrolizumab Nivolumab Both	25% (*n* = 4) 69% (*n* = 11) 6% (*n* = 1)	60% (*n* = 3) 40% (*n* = 2)	0.29

## Data Availability

De-identified data presented in this study are not publicly available but can be requested from the corresponding author.
